# 5-(4-Methyl­piperazin-1-yl)-2-nitro­aniline

**DOI:** 10.1107/S1600536810015953

**Published:** 2010-05-08

**Authors:** Chang-jun Luan, Cheng Guo, Wei Wang, Jian-qiang Wang, Ren-jun Du

**Affiliations:** a Department of Applied Chemistry, College of Science, Nanjing University of Technology, Nanjing 210009, People’s Republic of China

## Abstract

In the title compound, C_11_H_16_N_4_O_2_, the dihedral angle between the benzene ring and the plane of the four carbon atoms in the piperazine ring is 12.17 (3)°; the latter ring adopts a chair conformation.    An intramolecular N—H⋯O hydrogen bond generates an *S*(6) ring.  In the crystal, the molecules are linked by N—H⋯N hydrogen bonds, forming chains.

## Related literature

For bond-length data, see: Allen *et al.* (1987 ▶). For the synthetic procedure and use of the title compound as an inter­mediate in the synthesis of tyrosine kinase inhibitors, see: Renhowe *et al.* (2009 ▶).
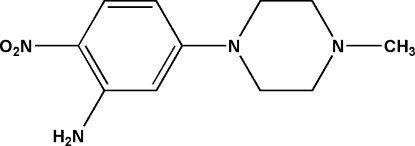


## Experimental

### 

#### Crystal data

C_11_H_16_N_4_O_2_
*M* *_r_* = 236.28
Monoclinic, 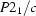

*a* = 11.027 (2) Å
*b* = 6.121 (1) Å
*c* = 17.524 (4) Å
β = 103.79 (3)°
*V* = 1148.7 (4) Å^3^
*Z* = 4
Mo *K*α radiation
μ = 0.10 mm^−1^
*T* = 293 K
0.30 × 0.20 × 0.05 mm

#### Data collection

Enraf–Nonius CAD-4 diffractometer
Absorption correction: ψ scan (North *et al.*, 1968 ▶) *T*_min_ = 0.971, *T*_max_ = 0.995
2205 measured reflections
2090 independent reflections
1358 reflections with *I* > 2σ(*I*)
*R*_int_ = 0.042
3 standard reflections every 200 reflections  intensity decay: 1%

#### Refinement

*R*[*F* ^2^ > 2σ(*F* ^2^)] = 0.064
*wR*(*F* ^2^) = 0.192
*S* = 1.01
2090 reflections
155 parameters
H-atom parameters constrained
Δρ_max_ = 0.25 e Å^−3^
Δρ_min_ = −0.18 e Å^−3^

### 

Data collection: *CAD-4 Software* (Enraf–Nonius, 1985 ▶); cell refinement: *CAD-4 Software*; data reduction: *XCAD4* (Harms & Wocadlo, 1995 ▶); program(s) used to solve structure: *SHELXS97* (Sheldrick, 2008 ▶); program(s) used to refine structure: *SHELXL97* (Sheldrick, 2008 ▶); molecular graphics: *SHELXTL* (Sheldrick, 2008 ▶); software used to prepare material for publication: *SHELXTL*.

## Supplementary Material

Crystal structure: contains datablocks I, global. DOI: 10.1107/S1600536810015953/im2192sup1.cif

Structure factors: contains datablocks I. DOI: 10.1107/S1600536810015953/im2192Isup2.hkl

Additional supplementary materials:  crystallographic information; 3D view; checkCIF report

## Figures and Tables

**Table 1:** Hydrogen-bond geometry (Å, °)

*D*—H⋯*A*	*D*—H	H⋯*A*	*D*⋯*A*	*D*—H⋯*A*
N3—H3*C*⋯N1^i^	0.86	2.39	3.156 (4)	148
N3—H3*D*⋯O1	0.86	2.06	2.669 (4)	127

Symmetry code: (i) 
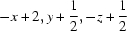
.

**Fig. 1.**
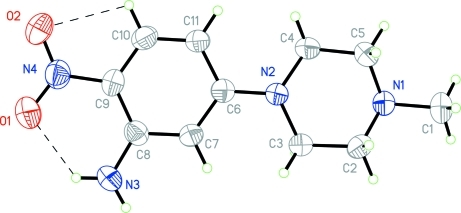
The molecular structure of (I), with the atom-numbering scheme. Displacement ellipsoids are drawn at the 50% probability level.

**Fig. 2.**
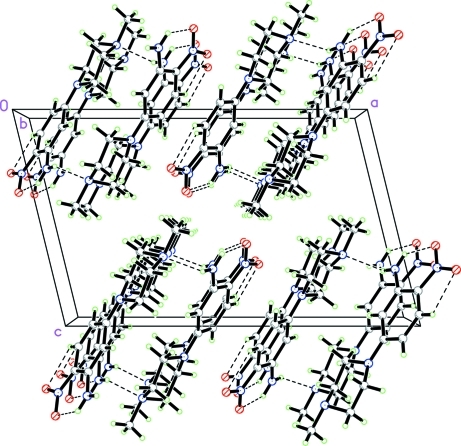
A packing diagram of (I). Hydrogen bonds are shown as dashed lines.

## supplementary crystallographic information

### Comment

The title compound, (I), has been reported as an intermediate for the synthesis of novel tyrosine kinase inhibitors (Renhowe, P. A. *et al.*, 2009). We herein report its crystal structure.

In the molecular structure of (I), (Fig.1), bond lengths (Allen *et al.*, 1987) and angles are within normal ranges. N2, N3 and N4 atoms are almost coplanar with the benzene ring to which they are bonded [deviations of 0.078 (1), 0.052 (1) and 0.078 (1) Å]. The plane of C2—C3—C4—C5 is nearly parallel with the benzene ring plane (the torsion angle is 12.17 (3) °). By contrast, due to the piperazine moiety adopting a chair conformation N1—C2—C5 and N2—C3—C4 form two separate planes with torsion angle of 45.87 (2) ° and 25.92 (3) °, respectively, with respect to the benzene ring. The crystal structure of the title compound exhibits N—H···O, C—H···O, and N—H···N intra- and intermolecular hydrogen bonds to form a three dimensional network.

As can be seen from the packing diagram, (Fig. 2), the molecules are stacked along the *b* axis.

### Experimental

The title compound, (I) was prepared by a literature method (Renhowe, P. A. *et al.*, 2009). Crystals suitable for X-ray analysis were obtained by dissolving (I) (0.5 g) in methanol (20 ml) and evaporating the solvent slowly at room temperature for about 7 d.

### Refinement

H atoms were positioned geometrically, with N—H = 0.86 Å, C—H = 0.93 Å for aromatic H, 0.97 Å for methylene and 0.96 Å for methyl groups. Refinement was performed using a riding model with *U*_iso_(H) = *xU*_eq_(C), where x = 1.5 for methyl and x = 1.2 for all other H atoms.

### Crystal data

**Table:**

C_11_H_16_N_4_O_2_	*D*_x_ = 1.366 Mg m^−^^3^
*M**_r_* = 236.28	Melting point: 428 K
Monoclinic, *P*2_1_/*c*	Mo *K*α radiation, λ = 0.71073 Å
*a* = 11.027 (2) Å	Cell parameters from 25 reflections
*b* = 6.121 (1) Å	θ = 9–13°
*c* = 17.524 (4) Å	µ = 0.10 mm^−^^1^
β = 103.79 (3)°	*T* = 293 K
*V* = 1148.7 (4) Å^3^	Block, yellow
*Z* = 4	0.30 × 0.20 × 0.05 mm
*F*(000) = 504	

### Data collection

**Table:**

Enraf–Nonius CAD-4 diffractometer	1358 reflections with *I* > 2σ(*I*)
Radiation source: fine-focus sealed tube	*R*_int_ = 0.042
graphite	θ_max_ = 25.3°, θ_min_ = 1.9°
ω/2θ scans	*h* = 0→13
Absorption correction: ψ scan (North *et al.*, 1968)	*k* = 0→7
*T*_min_ = 0.971, *T*_max_ = 0.995	*l* = −21→20
2205 measured reflections	3 standard reflections every 200 reflections
2090 independent reflections	intensity decay: 1%

### Refinement

**Table:**

Refinement on *F*^2^	Secondary atom site location: difference Fourier map
Least-squares matrix: full	Hydrogen site location: inferred from neighbouring sites
*R*[*F*^2^ > 2σ(*F*^2^)] = 0.064	H-atom parameters constrained
*wR*(*F*^2^) = 0.192	*w* = 1/[σ^2^(*F*_o_^2^) + (0.1*P*)^2^ + 0.3*P*] where *P* = (*F*_o_^2^ + 2*F*_c_^2^)/3
*S* = 1.01	(Δ/σ)_max_ < 0.001
2090 reflections	Δρ_max_ = 0.25 e Å^−^^3^
155 parameters	Δρ_min_ = −0.18 e Å^−^^3^
0 restraints	Extinction correction: *SHELXL97* (Sheldrick, 2008), Fc^*^=kFc[1+0.001xFc^2^λ^3^/sin(2θ)]^-1/4^
Primary atom site location: structure-invariant direct methods	Extinction coefficient: 0.038 (6)

### Special details

**Table:**

Geometry. All esds (except the esd in the dihedral angle between two l.s. planes) are estimated using the full covariance matrix. The cell esds are taken into account individually in the estimation of esds in distances, angles and torsion angles; correlations between esds in cell parameters are only used when they are defined by crystal symmetry. An approximate (isotropic) treatment of cell esds is used for estimating esds involving l.s. planes.
Refinement. Refinement of *F*^2^ against ALL reflections. The weighted *R*-factor wR and goodness of fit *S* are based on *F*^2^, conventional *R*-factors *R* are based on *F*, with *F* set to zero for negative *F*^2^. The threshold expression of *F*^2^ > σ(*F*^2^) is used only for calculating *R*-factors(gt) etc. and is not relevant to the choice of reflections for refinement. *R*-factors based on *F*^2^ are statistically about twice as large as those based on *F*, and *R*- factors based on ALL data will be even larger.

### Fractional atomic coordinates and isotropic or equivalent isotropic displacement parameters (Å^2^)

**Table:**

	*x*	*y*	*z*	*U*_iso_*/*U*_eq_	
N1	0.6711 (2)	0.0465 (4)	0.16875 (13)	0.0438 (6)	
O1	1.3775 (2)	−0.0656 (4)	0.56703 (14)	0.0774 (8)	
C1	0.5525 (3)	0.1090 (6)	0.11543 (19)	0.0603 (9)	
H1A	0.5683	0.2064	0.0760	0.090*	
H1B	0.5007	0.1809	0.1447	0.090*	
H1C	0.5107	−0.0193	0.0907	0.090*	
N2	0.8586 (2)	0.0019 (4)	0.31264 (13)	0.0397 (6)	
O2	1.2787 (2)	−0.3532 (4)	0.58981 (13)	0.0662 (7)	
C2	0.7330 (3)	0.2397 (5)	0.20898 (17)	0.0484 (8)	
H2A	0.6826	0.3007	0.2422	0.058*	
H2B	0.7409	0.3493	0.1705	0.058*	
C3	0.8606 (3)	0.1827 (5)	0.25841 (16)	0.0466 (8)	
H3A	0.9143	0.1440	0.2240	0.056*	
H3B	0.8960	0.3103	0.2883	0.056*	
N3	1.2683 (2)	0.2156 (4)	0.45524 (16)	0.0613 (8)	
H3C	1.2595	0.3315	0.4267	0.074*	
H3D	1.3359	0.1954	0.4909	0.074*	
C4	0.7741 (3)	−0.1801 (5)	0.28094 (18)	0.0478 (8)	
H4A	0.7577	−0.2645	0.3242	0.057*	
H4B	0.8149	−0.2755	0.2506	0.057*	
N4	1.2841 (2)	−0.1869 (5)	0.55073 (15)	0.0527 (7)	
C5	0.6513 (3)	−0.1026 (5)	0.22932 (17)	0.0506 (8)	
H5A	0.6035	−0.2276	0.2048	0.061*	
H5B	0.6033	−0.0292	0.2615	0.061*	
C6	0.9667 (2)	−0.0433 (4)	0.36840 (15)	0.0367 (7)	
C7	1.0669 (2)	0.1023 (5)	0.38534 (15)	0.0398 (7)	
H7A	1.0610	0.2307	0.3563	0.048*	
C8	1.1758 (2)	0.0647 (5)	0.44396 (16)	0.0415 (7)	
C9	1.1820 (2)	−0.1317 (5)	0.48727 (15)	0.0422 (7)	
C10	1.0839 (3)	−0.2817 (5)	0.46870 (17)	0.0475 (8)	
H10A	1.0901	−0.4120	0.4966	0.057*	
C11	0.9799 (3)	−0.2428 (5)	0.41112 (17)	0.0444 (7)	
H11A	0.9169	−0.3471	0.3995	0.053*	

### Atomic displacement parameters (Å^2^)

**Table:**

	*U*^11^	*U*^22^	*U*^33^	*U*^12^	*U*^13^	*U*^23^
N1	0.0385 (13)	0.0422 (14)	0.0464 (13)	−0.0004 (11)	0.0017 (10)	0.0030 (11)
O1	0.0545 (14)	0.0794 (18)	0.0820 (17)	−0.0125 (13)	−0.0158 (12)	0.0122 (14)
C1	0.0411 (17)	0.067 (2)	0.065 (2)	0.0050 (16)	−0.0025 (15)	0.0064 (18)
N2	0.0357 (12)	0.0346 (12)	0.0458 (13)	−0.0029 (10)	0.0040 (10)	0.0048 (11)
O2	0.0610 (15)	0.0616 (15)	0.0674 (15)	0.0125 (12)	−0.0015 (12)	0.0190 (12)
C2	0.0527 (18)	0.0375 (16)	0.0502 (17)	0.0004 (14)	0.0030 (14)	0.0085 (14)
C3	0.0449 (17)	0.0396 (16)	0.0503 (17)	−0.0067 (14)	0.0016 (14)	0.0084 (14)
N3	0.0486 (15)	0.0509 (16)	0.0724 (17)	−0.0149 (13)	−0.0094 (13)	0.0073 (14)
C4	0.0440 (16)	0.0354 (15)	0.0605 (18)	−0.0053 (13)	0.0057 (14)	0.0075 (14)
N4	0.0484 (15)	0.0537 (17)	0.0516 (15)	0.0041 (14)	0.0033 (12)	0.0020 (13)
C5	0.0383 (16)	0.0456 (17)	0.0636 (19)	−0.0060 (14)	0.0036 (14)	0.0058 (16)
C6	0.0354 (14)	0.0369 (15)	0.0393 (14)	0.0018 (12)	0.0118 (12)	−0.0007 (12)
C7	0.0414 (15)	0.0320 (15)	0.0443 (15)	0.0013 (12)	0.0071 (12)	0.0025 (12)
C8	0.0391 (15)	0.0386 (16)	0.0455 (16)	−0.0015 (13)	0.0075 (13)	−0.0057 (13)
C9	0.0398 (15)	0.0469 (17)	0.0377 (15)	0.0063 (13)	0.0051 (12)	0.0029 (13)
C10	0.0470 (17)	0.0444 (18)	0.0510 (17)	0.0014 (14)	0.0116 (14)	0.0123 (14)
C11	0.0391 (15)	0.0395 (16)	0.0527 (17)	−0.0031 (13)	0.0071 (13)	0.0101 (14)

### Geometric parameters (Å, °)

**Table:**

N1—C5	1.455 (3)	N3—H3C	0.8600
N1—C2	1.460 (4)	N3—H3D	0.8600
N1—C1	1.466 (3)	C4—C5	1.514 (4)
O1—N4	1.246 (3)	C4—H4A	0.9700
C1—H1A	0.9600	C4—H4B	0.9700
C1—H1B	0.9600	N4—C9	1.422 (4)
C1—H1C	0.9600	C5—H5A	0.9700
N2—C6	1.377 (3)	C5—H5B	0.9700
N2—C3	1.462 (3)	C6—C7	1.395 (4)
N2—C4	1.473 (3)	C6—C11	1.421 (4)
O2—N4	1.236 (3)	C7—C8	1.401 (4)
C2—C3	1.507 (4)	C7—H7A	0.9300
C2—H2A	0.9700	C8—C9	1.415 (4)
C2—H2B	0.9700	C9—C10	1.396 (4)
C3—H3A	0.9700	C10—C11	1.356 (4)
C3—H3B	0.9700	C10—H10A	0.9300
N3—C8	1.355 (3)	C11—H11A	0.9300
			
C5—N1—C2	106.8 (2)	N2—C4—H4B	109.1
C5—N1—C1	111.2 (2)	C5—C4—H4B	109.1
C2—N1—C1	109.8 (2)	H4A—C4—H4B	107.8
N1—C1—H1A	109.5	O2—N4—O1	120.6 (3)
N1—C1—H1B	109.5	O2—N4—C9	119.7 (3)
H1A—C1—H1B	109.5	O1—N4—C9	119.7 (3)
N1—C1—H1C	109.5	N1—C5—C4	111.3 (2)
H1A—C1—H1C	109.5	N1—C5—H5A	109.4
H1B—C1—H1C	109.5	C4—C5—H5A	109.4
C6—N2—C3	118.0 (2)	N1—C5—H5B	109.4
C6—N2—C4	118.6 (2)	C4—C5—H5B	109.4
C3—N2—C4	115.7 (2)	H5A—C5—H5B	108.0
N1—C2—C3	110.8 (2)	N2—C6—C7	122.0 (2)
N1—C2—H2A	109.5	N2—C6—C11	120.6 (2)
C3—C2—H2A	109.5	C7—C6—C11	117.4 (2)
N1—C2—H2B	109.5	C6—C7—C8	123.2 (3)
C3—C2—H2B	109.5	C6—C7—H7A	118.4
H2A—C2—H2B	108.1	C8—C7—H7A	118.4
N2—C3—C2	113.1 (2)	N3—C8—C7	118.6 (3)
N2—C3—H3A	109.0	N3—C8—C9	124.2 (2)
C2—C3—H3A	109.0	C7—C8—C9	117.2 (2)
N2—C3—H3B	109.0	C10—C9—C8	119.9 (2)
C2—C3—H3B	109.0	C10—C9—N4	116.8 (3)
H3A—C3—H3B	107.8	C8—C9—N4	123.3 (3)
C8—N3—H3C	120.0	C11—C10—C9	121.9 (3)
C8—N3—H3D	120.0	C11—C10—H10A	119.0
H3C—N3—H3D	120.0	C9—C10—H10A	119.0
N2—C4—C5	112.5 (2)	C10—C11—C6	120.3 (3)
N2—C4—H4A	109.1	C10—C11—H11A	119.8
C5—C4—H4A	109.1	C6—C11—H11A	119.8
			
C5—N1—C2—C3	64.5 (3)	C6—C7—C8—N3	179.3 (3)
C1—N1—C2—C3	−174.7 (2)	C6—C7—C8—C9	−0.1 (4)
C6—N2—C3—C2	−170.8 (2)	N3—C8—C9—C10	−177.0 (3)
C4—N2—C3—C2	40.4 (3)	C7—C8—C9—C10	2.4 (4)
N1—C2—C3—N2	−53.1 (3)	N3—C8—C9—N4	3.6 (4)
C6—N2—C4—C5	171.8 (2)	C7—C8—C9—N4	−177.1 (2)
C3—N2—C4—C5	−39.5 (3)	O2—N4—C9—C10	−5.1 (4)
C2—N1—C5—C4	−64.2 (3)	O1—N4—C9—C10	175.8 (3)
C1—N1—C5—C4	175.9 (3)	O2—N4—C9—C8	174.4 (3)
N2—C4—C5—N1	52.0 (3)	O1—N4—C9—C8	−4.8 (4)
C3—N2—C6—C7	13.8 (4)	C8—C9—C10—C11	−1.8 (4)
C4—N2—C6—C7	161.8 (2)	N4—C9—C10—C11	177.7 (3)
C3—N2—C6—C11	−166.2 (2)	C9—C10—C11—C6	−1.2 (5)
C4—N2—C6—C11	−18.2 (4)	N2—C6—C11—C10	−176.7 (3)
N2—C6—C7—C8	177.3 (2)	C7—C6—C11—C10	3.3 (4)
C11—C6—C7—C8	−2.7 (4)		

### Hydrogen-bond geometry (Å, °)

**Table:**

*D*—H···*A*	*D*—H	H···*A*	*D*···*A*	*D*—H···*A*
N3—H3C···N1^i^	0.86	2.39	3.156 (4)	148
N3—H3D···O1	0.86	2.06	2.669 (4)	127
C10—H10A···O2	0.93	2.35	2.671 (4)	100

Symmetry codes: (i) −*x*+2, *y*+1/2, −*z*+1/2.
